# Influence of Microstructure on Music Properties of SWP-B Music Steel Wire Under Different Annealing Treatments

**DOI:** 10.3390/ma18020440

**Published:** 2025-01-18

**Authors:** Xinru Jia, Qinghua Li, Fuguo Li, Xiaohui Fang, Junda You, Qian Zhao, Xia Wang, Jinhua Lu

**Affiliations:** 1 State Key Laboratory of Solidification Processing, School of Materials Science and Engineering, Northwestern Polytechnical University, Xi’an 710072, China; jiaxinru@mail.nwpu.edu.cn (X.J.); fxh@mail.nwpu.edu.cn (X.F.); yjd@mail.nwpu.edu.cn (J.Y.); zq2019@mail.nwpu.edu.cn (Q.Z.); 2 Shaanxi Key Laboratory of High-Performance Precision Forming Technology and Equipment, Northwestern Polytechnical University, Xi’an 710072, China; 3 Joint R&D Center for Metallic Materials, Metallic Wire and Metallic Card Clothing, Xi’an 710021, China; 4 Department of Engineering Mechanics, College of Civil & Architecture Engineering, Xi’an Technological University, Xi’an 710021, China

**Keywords:** music steel wire, microstructure, cementite spheroidization, spectral properties, musical expression

## Abstract

The mechanical properties of music wire are contingent upon its microstructure, which in turn influences its applications in music. Chinese stringed instruments necessitate exacting standards for comprehensive performance indexes, particularly with regard to the strength, resilience, and rigidity of the musical steel wires, which differ from the Western approach to musical wire. In this study, SWP-B music wire was selected for investigation through metal heat treatment, which was employed to regulate its microstructure characteristics. Furthermore, a spectral analysis was conducted to evaluate the musical expression, encompassing attributes such as pitch and timbre. In conclusion, the governing law of the impact of the microstructure of music wire on its musical expression was established. The results demonstrate that steel wire subjected to a 200 °C annealing treatment for cementite spheroidization can effectively reduce stress concentration, thereby reducing the probability of fracture and consequently improving tonal uniformity and richness while increasing tensile strength from 2578 MPa to 2702 MPa. Conversely, the high-temperature annealing treatment alters the crystalline structure of the material and refines the grain structure, thereby improving the material’s performance and sound quality. The fine microstructure of the music steel wire displays enhanced uniformity. As the annealing temperature increases, the strength of the ferrite phase <110>//ND (<010>//ND, indicating that the <010> direction of the crystal is parallel to the normal direction of the material) and the cementite phase <010>//ND demonstrates a gradual decline. However, this also results in a more pronounced harmonic performance, which, in turn, affects the overall music expression.

## 1. Introduction

As an important component of stringed instruments, the evolution of music steel wire reflects the close integration of music and materials engineering. From the initial utilization of natural materials, such as sheep gut strings, to the advent of modern high-performance steel wires, the evolution of stringed instruments not only aligns with the trajectory of technological advancement but also exemplifies the uninterrupted enhancement of musical expression. In classical Chinese music, each instrument can express a unique set of emotions. The guqin, a seven-stringed plucked instrument in some ways similar to the zither, is an instrument that can convey profound emotions in a reserved and distant manner. The erhu (two-string fiddle) is particularly adept at expressing sadness and tenderness, rendering it an appropriate instrument for conveying delicate emotions. The pipa (pear-shaped lute/Chinese lute), with its dexterous fingering and variable speed, is a highly expressive musical instrument. As music steel wire has become more widely used in musical instruments, the tone of these instruments has become brighter, allowing them to meet the diverse demands of modern musical expressions. The advancement of classical Chinese musical instruments and the evolution of string materials reflect the ongoing transformation of musical culture, while simultaneously illustrating the multifaceted nature of musical expression. The advent of music steel wire represents a pivotal moment in this evolution, enabling traditional instruments to flourish once more in the context of contemporary music.

The selection of material for music steel wire is primarily based on its acoustic characteristics, including pitch accuracy, low internal damping, high elasticity, and toughness. In addition to these acoustic properties, cost and durability are also important considerations. The forming process exerts a significant influence on the properties of music steel wire. A variety of drawing and processing techniques can be employed to produce strings with varying diameters and properties. In recent years, research on forming processes has focused on the optimization of drawing speed, annealing temperature, and cooling method. For example, the implementation of continuous drawing and multistage annealing processes has been demonstrated to enhance the uniformity and elasticity of strings. From the perspective of practical engineering, drawn steel wires are subjected to additional heat treatment during the manufacturing process. Such processes may include bluing at 300 °C to 400 °C or hot-dip galvanizing at 450 °C [1,2,3]. These temperatures are high enough to modify the mobility of dislocations and alloying elements within the deformation structure, thereby influencing the mechanical properties of the material. In light of these considerations, significant effort has been dedicated to elucidating the effects of annealing on the deformation microstructure [4]. Li et al. observed that, during high-temperature annealing, spheroidized cementite particles were situated at triple junctions between coarse hexagonal ferrite substructure grains [2]. The findings revealed that the mechanical properties of strings are markedly influenced by the specific heat treatment parameters. In this field, a number of heat treatment protocols have been proposed for different materials. For example, a variety of heat treatment techniques have been proposed for high-carbon and alloy steels with the objective of attaining the most favorable balance between strength and modulus of elasticity. The advent of high-quality stainless steel and alloy materials has resulted in a notable enhancement in the toughness, stability, and acoustic quality of the musical steel wire [5].

Modifications to the microstructure of music wire have a direct impact on its modulus of elasticity, yield strength, fatigue life, and acoustic properties, which subsequently influence the range of frequencies and timbre it is capable of producing [6]. The presence of finer grains typically results in enhanced material strength and resilience. Furthermore, the anisotropy of the metal is closely associated with its crystal orientation. An optimal crystal orientation facilitates enhanced sound conduction efficiency and sound quality. Recent studies have demonstrated that the phase composition and interactions within the microstructure of music steel wire exert a significant influence on determining the material’s acoustic properties of the material. This is illustrated by the observation that grain refinement can enhance the strength and elasticity of the steel wire [7]. In the event of high strain, cementite is observed to undergo chemical decomposition and act as a carbon source through mechanical alloying. This process results in the segregation of carbon to ferrite boundaries, thereby enhancing the tensile strength of the material [8,9]. The optimization of the microstructure through heat treatment and forming processes has been demonstrated to enhance the fatigue resistance and the service life of the strings. Zhou et al. observed that annealing at temperatures ranging from 210 °C to 350 °C did not alter the morphology of cementite in the wire, but did result in a structural change in the cementite from an almost amorphous state to a nanocrystalline form.

The steel wire utilized in musical instruments is subjected to meticulous regulation with regard to a multitude of factors, including surface finish, tensile strength, stiffness, and other mechanical and fatigue-related properties [10]. The mechanical properties of music steel wire strings serve as the fundamental indicators of their suitability for performance. The evaluation of string performance necessitates the consideration of a number of pivotal parameters, including tensile strength, fatigue resistance, and Young’s modulus. The mechanical properties of the material are largely contingent upon the dimensions and configuration of the pearlitic lamellae. In general, a reduction in the spacing of the pearlitic lamellae results in an increase in the yield strength, tensile strength, hardness, and elongation of the material [11]. The optimal layer spacing is conducive to optimizing the wire’s vibration characteristics, thereby enhancing the stability of pitch and the clarity of sound. An excessive layer spacing can result in an uneven tonal response of the wire during vibration, whereas a smaller layer spacing helps to produce a more uniform sound.

The expressiveness of music steel wire is contingent upon three primary factors: timbre, tone intensity, and range. An acoustic analysis revealed that the timbre and tone intensity of a given instrument can be significantly influenced by the material from which it is constructed and the characteristics of its microstructure. These factors can consequently affect both the performance of the player and the experience of the listener. Hidekazu Kodama conducted a comprehensive analysis of the impact of diverse string materials on timbre and performance characteristics, uncovering notable discrepancies in resonance frequency, sound quality, and aftertaste across different string materials. In particular, the elasticity and density of the strings exert a direct influence on the characterization of the sound propagation [12]. Jonathan Paul Crosmer conducted a comprehensive analysis of the factors influencing the quality of string tone, integrating performance tests, recordings, and harmonic frequency analysis. His findings revealed that the primary determinants of string tone quality are the composition and tension of the strings, the type of instrument being used, vibrations transmitted from the strings, bow speed, bow pressure, and the contact point between the bow and the strings [13].

The tension in strings is typically increased in order to achieve a more robust sound, but this is limited by the tensile strength of the music steel wire. There are three kinds of common steel wires used for strings: the first category, designated as SWP-A, exhibits a diameter range of 0.08 mm to 10.0 mm. These wires find primary application in general dynamic load springs, encompassing various types of pressure, tension, and torsion springs. The second category, designated as SWP-B, features a diameter range of 0.08 mm to 8.00 mm, suitable for dynamic load springs, with a relatively higher working stress, commonly used in automobile engine valve springs, precision instruments, electrical equipment, production machinery, brake springs, clutch springs, refrigerator compressor springs, etc. SWP-V, with a diameter range of 1.00~6.00 mm, is used specifically for valve springs or similar applications with higher requirements for springs. For a given material, the only means of increasing the available tension is to increase the diameter of the wire [14]. To augment the available tension for a given string material, it is essential to augment the diameter of the music steel wire. Nevertheless, an increase in wire diameter precipitates an enhancement of the bending stiffness of the wire. Excessive bending stiffness of the strings can have a deleterious impact on the sound quality [15,16]. The impact of string stiffness on the timbre and tuning methodology of the instrument is well documented [17]. All genuine strings exhibit a specific degree of stiffness, which is contingent upon the diameter and other intrinsic material properties of the string. It has been demonstrated that the discordance of the vibration of the music steel wire can be reduced when the tension is increased to the ultimate tensile strength (*T_S_*), as shown in the following equations:

(1) $$f_{n}=n\cdot f_{1}(1+\alpha \cdot n^{2})$$

(2) $$\alpha =\frac{\pi^{3}\cdot Er^{4}}{8L^{2}T}$$

In Equation (1), $f=\sqrt{c/L}$ is the fundamental frequency of the string (here *c* is the wave velocity, $c=\sqrt{T/\rho }$, *L* is the effective vibration length of the string, and *ρ* is the linear density of the string). *α* is the dissonance factor (the deviation from the harmonic is measured by the value of the dissonance factor *α*), *T* is the tensile force, and *Er* is the Young’s modulus. An increase in the ultimate tensile strength of the music steel wire allows for the creation of smaller dissonances [18]. The enhanced strength and ductility of the wire enable it to withstand higher tensile forces without rupturing, thereby increasing its lifespan while maintaining optimal elasticity. This quality contributes to pitch stability and consistent aural quality throughout the playing process. Additionally, the use of this wire in industrial production settings can result in cost savings, making it a cost-effective solution for industrial applications.

A substantial body of research has been conducted on the microstructure of steel wires subjected to heat treatment. Nevertheless, the microstructural evolution of steel wires during the annealing process, as well as the correlation between the changes in tensile strength and their relationship to music steel wires, remain unclear. In this study, the spectral information of Fourier-transformed sound waves generated by the vibration of string wires subjected to different annealing treatments is employed to investigate the musical expression. Scanning electron microscopy (SEM), X-ray diffraction (XRD), and electron backscatter diffraction (EBSD) were employed to investigate the microstructure of music steel wire in diverse annealing conditions. The transformation in mechanical properties of music steel wire at varying annealing temperatures was delineated through the utilization of nanoindentation hardness and tensile properties. Furthermore, the Fourier transform of sound waves generated by the vibration of music steel wire was analyzed using MATLAB R2023b to examine the vibration characteristics and spectral response of the wire. Additionally, the parameters of Young’s modulus, tensile strength, nanoindentation hardness, and decay time of the music steel wire were measured and calculated, respectively, to analyze the musical music expression parametrically from three perspectives: tone, amplitude, and loudness. Moreover, the impact of varying microstructures, mechanical properties, and string wrapping on the musical music expression of the music steel wire was evaluated. A comprehensive analysis of the large-strain SWP-B music wire was conducted through additional annealing of cementite spheroidization, ferrite deformation, and other phenomena, with the aim of systematically exploring the relationship between mechanical properties and string composition, manufacturing process, and mechanical properties of music steel wire. This was accomplished through an examination of the music steel wire following the annealing of disparate internal cementite spheroidization processes. The findings indicate that the internal cementite spheroidization of music steel wire has a considerable impact on the musical expression, thereby advancing the existing conclusions.

## 2. Experimental Materials and Methods

**Figure 1:**
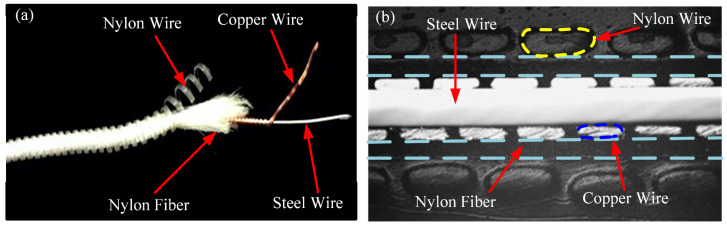
(**a**) String breakdown diagram; (**b**) String longitudinal section.

**Figure 2:**
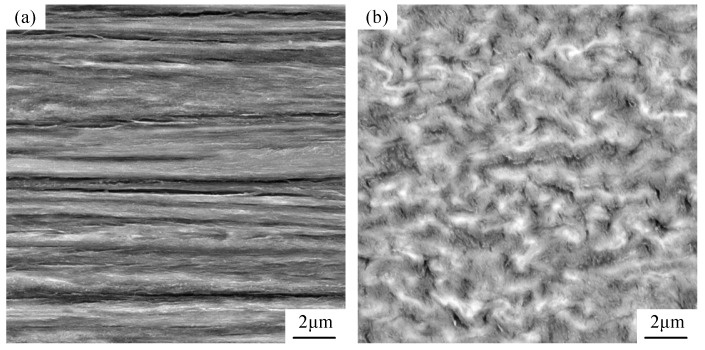
(**a**) Longitudinal section SEM graph of the music wire; (**b**) Cross-section SEM graph of the music wire.

**Figure 3:**
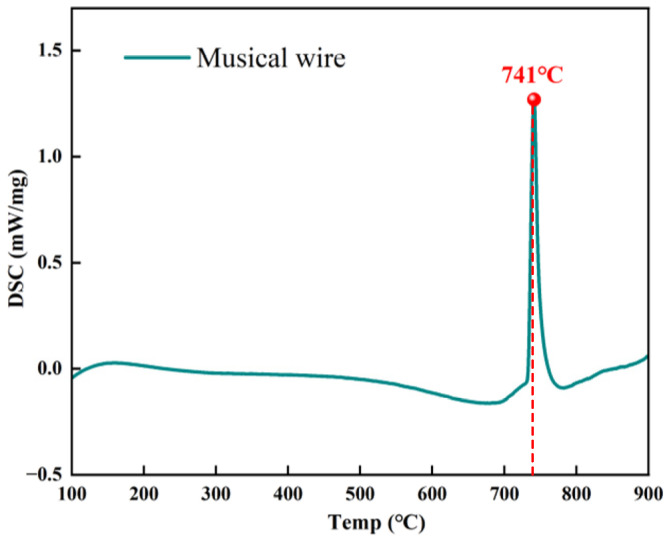
The phase transition curve for DSC of SWP-B music wire.

The materials employed in this experiment were guzheng strings procured from the Xi’an Conservatory of Music Musical Instrument Factory. The guzheng is one of the oldest folk musical instruments in China. It is a 21- or 25-stringed plucked instrument. The strings were stripped of the outer coil wire, nylon wire, and copper wire. The resulting decomposition diagram is illustrated in Figure 1a, while the sectional diagram is shown in Figure 1b. The steel wire located within the core was retained for the purpose of undergoing experimental testing, and its designated model is SWP-B. The initial microstructure of the music steel wire is illustrated in Figure 2. The microstructure comprises layered pearlitic structures, comprising ferrite and cementite, which are markedly elongated along the axes of the strings and exhibit a fine fiber texture. The chemical composition of the music steel wire was determined in accordance with the Chinese Industrial Standard YB/T 5218-2017, as detailed in Table 1. A differential scanning calorimetry (DSC) test was conducted on the music steel wires using a simultaneous thermal analyzer (STA 449C) from NETZSCH (Selb, Germany), as shown in Figure 3, and the peak of the heat flow was identified within the range of approximately 700–800 °C, indicating that the phase transition point of the music steel wires is 741 °C.

The elemental composition of high-carbon steel wire is of the utmost importance, particularly the carbon content. Steel wire exhibits a strong magnetic property and, due to the low atomic number of carbon and sulfur, cannot be scanned accurately using an energy dispersive spectrometer (EDS) electronic probe. This paper utilizes the CS844 Carbon and Sulfur Analyzer (LECO, St. Joseph, MO, USA) to measure the carbon and sulfur content. The method is based on the standard GB/T 20123-2006, which details the determination of the total carbon and sulfur content of iron and steel. The combustion of the sample after being poured into the infrared detection cell is performed using a high-frequency induction furnace, and the infrared absorption method is used to calculate the content. This method ensures high accuracy. A UV–visible near-infrared spectrophotometer is employed to measure the silicon, manganese, and phosphorus content. The sample is dissolved in acid and subsequently reacted with reagents to create color. The measurement of the sample’s absorption at distinct wavelengths enables the calculation of each element’s content.

### 2.1. Microstructure and Mechanical Property Test

A differential scanning calorimetry (DSC) test was conducted on the music steel wires using a simultaneous thermal analyzer (STA449C) from NETZSCH (Selb, Germany) to perform the DSC thermal analysis of SWP-B wires. The tests were conducted using an aluminum crucible with a diameter of 4 mm and a crimp seal. Prior to the commencement of the DSC analysis, the ultra-fine steel wire was meticulously divided into segments of 3 mm in length. This procedure ensured that the aluminum crucible and the wire’s test surface were thoroughly devoid of any extraneous contaminants. The weight of the steel wire sample was then meticulously regulated within the range of 30 to 50 mg using an analytical balance. Subsequent to this, the aluminum crucible underwent a process of compaction through the utilization of a press. During the test, the heating rate was set to 20 K/min, the empty aluminum pan was kept at 40 °C for 1 min, and then the SWP-B steel wire was placed into the pan. The temperature interval was set from 40 °C to 900 °C. The utilization of flowing nitrogen as a protective gas during the course of the tests is also a notable aspect of the experimental design. The collected DSC test data were then subjected to thorough analysis using NETZSCH Proteus software 4.2.1. Heat flow was measured in relation to time and can be converted linearly to temperature. Heat flow in the positive lead was obtained by subtracting the heat flow in the pan from the heat flow in the pan and the lead.

The string steel wire has been subjected to a heat treatment process. This process effectively enhances the wire’s toughness and elasticity, thereby ensuring its stability under tension. It is distinguished by high tensile strength and high hardness. In this study, the music steel wire was subjected to a low-temperature annealing treatment at 100 °C, 150 °C, 200 °C, 250 °C, 300 °C, 350 °C, 400 °C, 450 °C, and 500 °C at temperatures below the phase transition point, with a holding time of 30 min.

**Figure 4:**
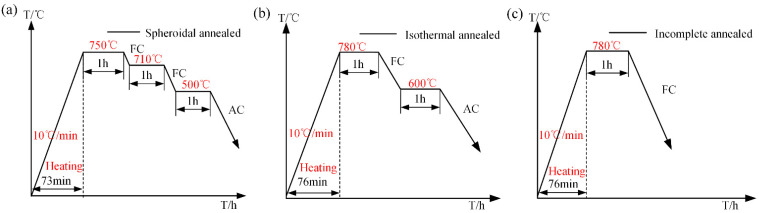
Annealing heat treatment curves of music wire: (**a**) Spheroidal annealing curve; (**b**) Isothermal annealing curve; (**c**) Incomplete annealing curve (FC: furnace cooling; AC: air cooling).

Given the high carbon content of music steel wire, it can be classified as hypereutectoid steel wire. Consequently, spheroidization annealing, isothermal annealing, and incomplete annealing were employed for music steel wires above the phase transition point. The resulting heat treatment curve is illustrated in Figure 4. Spheroidal annealing, as illustrated in Figure 4a, involves heating the steel wire to 750 °C, maintaining this temperature for one hour, and then the wire is cooled in the furnace to 710 °C (FC). This temperature is maintained for one hour, after which the furnace is used to cool the wire to 500 °C for one hour. Finally, it is removed from the furnace and cooled in air to room temperature (AC). To ensure uniformity in the spheroidization microstructure and precise control over the hardness of the annealed material, the objective is to achieve a uniform distribution of spherical or granular carbides within the ferrite matrix. Isothermal annealing is illustrated in Figure 4b: the music steel wire is heated to 780 °C for one hour to achieve incomplete austenitizing. It is then cooled more rapidly to 600 °C (the pearlitic temperature range) for one hour in an isothermal holding process. This allows the austenite to be completely transformed into pearlite. The material is then air-cooled to room temperature and isothermally annealed. The resulting microstructure and hardness are more uniform than those achieved through complete annealing. Incomplete annealing (Figure 4c) involves heating the music steel wire to 780 °C, maintaining the temperature for one hour to achieve incomplete austenitization, and then slowly cooling the wire to room temperature. This process can refine the microstructure and reduce the hardness of the material. The evolution of the microstructure and mechanical properties of the annealed music steel wires and the respective differences in the corresponding musical expression were observed and analyzed.

To examine the microscopic morphology of music steel wire following annealing treatment at varying temperatures, the heat-treated samples were inlaid. The music steel wire with a diameter of 0.5 mm and a length of 15 mm was affixed to the XQ-2B metallographic specimen inlay machine with double-sided adhesive tape from Shanghai Optical Machinery Factory. Thereafter, the wire was embedded with conductive resin and powder, resulting in a cylindrical sample with a diameter of 20 mm and a length of 10 mm. Once the resin had cured, polishing was conducted along the longitudinal axis of the wire, with the objective of achieving a polished surface that was as close as possible to the central axis and a width of the polished sample surface greater than 96% of the wire diameter. Subsequently, the samples were ground with sandpaper in a coarse-to-fine sequence, beginning with 1000# and progressing to 3000#. Following grinding, the samples were subjected to fine polishing with W2.5 diamond polishing paste on a tweed polishing cloth until all the scratches on the surface of the samples were removed, resulting in a bright surface. Subsequently, the samples were etched with a 3~4% nitric acid solution for 30s to observe the microstructure.

In this experiment, a metallographic optical microscope (Axiovert 40 MAT, Carl Zeiss, Oberkochen, Germany) was used to observe the metallographic microstructure of the music steel wire after corrosion at a magnification of 1000. Additionally, an ultra-high-resolution field emission scanning electron microscope (Clara GMH, Tescan, Czech Republic) with an electron-beam acceleration voltage of 15–20 kV was employed to observe the microstructure of the heat-treated music steel wire for SEM analysis. Finally, ImageJ2, an open Java image processing and analysis program, was used to observe the microstructure of the heat-treated music steel wire. The volume percentage of ferrite/pearlitic lamella spacing was determined from multiple optical micrographs using ImageJ2. Additionally, various crucial parameters, such as lamella spacing, were obtained from several high-resolution FE-SEM images through the linear intercept method. A field emission scanning electron microscope (ZEISS Sigma 300, Carl Zeiss, Oberkochen, Germany) equipped with a Nordlys Nano Detector was employed to obtain the Kikuchi pattern (TKD) of the music steel wire. Subsequently, the polar plots were generated using Aztec Crystal 2.1.2, EBSD analysis software, to analyze the textile structure orientation and grain size changes of the music steel wire after heat treatment.

In this experiment, X-ray diffractometer (D8 DISCOVER, Bruker, Billerica, MA, USA) was utilized to obtain the diffraction peak data of SWP-B steel wire for XRD analysis. The incoming ray method generator was the most common Cu target (λ = 0.1542 nm) with a wavelength of λ = 1.5406 Å. Prior to instrument use, calibration was conducted using a Si standard. It is imperative to note that since XRD is performed by scanning an area to obtain the diffraction peaks, it is essential to ensure that the area of the specimen is larger than 10 mm × 10 mm. The experiment was conducted with a tube voltage U = 40 kV, a tube current I = 200 mA, and a diffraction angle of 2θ in the scanning range of 30° to 130°.

In this experiment, the objective was to ascertain the impact of distinct annealing treatments on the hardness of music steel wire. Nanoindentation hardness testing (HIT) was conducted using a nanoindentation instrument (Hysitron TI 980, Bruker, Billerica, MA, USA) equipped with a Berkovich indenter, crafted from diamond with a radius of 100 nm. An A3 × 3 matrix of nanoindentation points was collected from the center to the edges of the longitudinal section of the mirror-polished music steel wire samples with a uniform spacing of 30 μm, forming a grid-like pattern. A load of 10 mN was applied, and the loading, holding, and unloading times were set to 5 s, 2 s, and 5 s, respectively. The final hardness values were averaged. Nanoindentation hardness was converted to Vickers hardness in accordance with the standards set forth in ISO 14577-1:2015(E), where 1 GPa is equivalent to 102.04 HV.

The objective of this study is to examine the relationship between alterations in microstructure and mechanical properties, with a particular focus on the tensile strength of music steel wire, which is a critical factor in determining its service life. As the diameter of music steel wire is insufficient for easy clamping, a tensile testing machine (INSTRON 3382, Boston, MA, USA) was employed to ascertain the tensile strength and Young’s modulus of music steel wire following various annealing treatments. The wire was twisted and affixed to fixtures at both ends, with node readings recorded at a step size of 10 N. The data were collected from multiple measurements, and the final fitting of the force and displacement change curves was converted into stress–strain curves. To ensure the reliability of the results, the data from the five tensile tests conducted on the music steel wires after each annealing condition were averaged.

### 2.2. Music Expression Test

In order to conduct a visual analysis of the musical expressiveness of the guzheng’s music steel wires, excitation was applied to the four steel wires with different heat treatments with the objective of inducing vibration. To record the vibration frequencies of the music steel wires in their playing state, an audio recording device was positioned near the tensioned music steel wires, with the objective of capturing the emitted sound frequencies. The audio recording device employed was a Multi-Track Linear PCM Recorder (LS-100, Olympus, Tokyo, Japan), which is capable of high-fidelity PCM recording at a maximum frequency of 96 kHz and a maximum of 24 bits. Following the excitation of the strings, the audio signals were recorded and the requisite program code was written in MATLAB software R2023b for the calculation of the Fourier transform (FFT) spectra of the acoustic signals. This was performed in order to analyze the frequency domain and time domain characteristics of the music steel wires after different treatments, as described in references [19,20]. The vibration of the string can be classified into four main categories: transverse vibration, longitudinal vibration, torsional vibration, and octave vibration. As the transverse vibration represents the primary form of vibration, this paper will concentrate on it. The fundamental vibration frequency formula proposed by Mason can be used to calculate the transverse vibration [21]:

(3) $$f=\frac{1}{2L}\sqrt{\frac{T}{\rho_{l}}}$$

In this equation, *T* represents tension (N), *L* denotes the effective string length (m), and the string’s line density is represented by $\rho_{l}$ (kg/m), $\rho_{l}$ = $\rho_{v}$ A = $\rho_{v}$ (πd^2^)/4. The density of the steel wire string (density of high-carbon steel wire) is 7850 kg/m^3^. It can be observed that the frequency of the transverse vibration is inversely proportional to the length of the string, directly proportional to the square root of the tension, and inversely proportional to the square root of the wire density.

The duration of an instrument’s sound signal articulation is significantly influenced by the decay time of its fundamental frequency. The term “decay time” is defined as the time required for the maximum amplitude of the fundamental frequency harmonic to decay to 10% of the maximum amplitude. This value is employed in the calculation of the duration of the interval of the fundamental frequency component of the sound signal generated by the vibration of the strings under disparate plucking strengths. The formula for this calculation is as follows:

(4) $$T=|T_{LPmax}-T_{LPmax\cdot 0.1}|$$

where $T_{LPmax}$ is the time from the onset of oscillation of the fundamental frequency signal to its maximum amplitude, $T_{LPmax\cdot 0.1}$ is the time for the fundamental frequency signal to decay from peak to 10% maximum amplitude.

**Figure 5:**
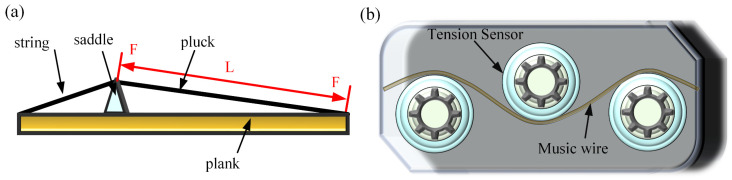
(**a**) Simple model of music wire oscillation; (**b**) Three-roller tension sensor measuring scheme. (Note: F: tension; L: length).

The tension of each string of the guzheng (simplified model in Figure 5a) was measured using a combination of the model DYZL-107 string tension tester and the three-roller tension sensor DY220-K1T2, manufactured by Anhui Bengbu Dayang Sensing Co., Ltd. (Bengbu, China) (see Figure 5b). The tension of the various heat-treated music steel wires was adjusted on a manual tensile tester (K-100HB-H, Fuzhou Aipu Instrument Co., Fuzhou, China), and the vibration frequency was subsequently measured after the application of excitation at varying tensions (*T*) and different vibration lengths (*L*). Subsequently, the audio data underwent Fourier transform analysis using MATLAB software R2023b, resulting in the generation of frequency domain and time domain plots of the music steel wires.

## 3. Experimental Results and Discussion

### 3.1. Microstructure Analysis of Music Wire After Low-Temperature Annealing

**Figure 6:**
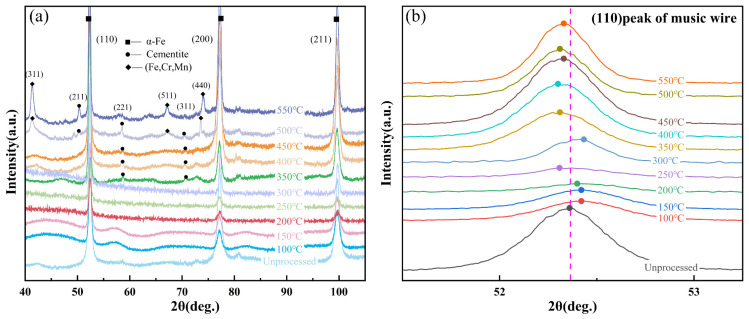
XRD patterns of music wires after different annealing treatments: (**a**) X-ray diffraction patterns; (**b**) Ferrite (110) crystal plane diffraction peaks.

Figure 6 shows the XRD patterns of music steel wires and various post-annealing treatments. The calibration of the indices for ferrite is based on card number PDF#06-0696, while that for cementite is based on card number PDF#34-0001. Figure 6a depicts the XRD patterns of music steel wire in various annealed states, whereas Figure 6b illustrates the ferrite (110) crystal diffraction peaks of the wire in various annealed states.

As shown in Figure 6a, at temperatures below 300 °C, the annealed music steel wire does not exhibit the diffuse cementite phase. Instead, C atoms are dissolved in the ferrite. As the annealing temperature increases, cementite is gradually precipitated. At 400 °C, precipitation of the Cr and Mn phases occurs. The diffraction peaks of the ferrite (110) appeared shifted, broadened, and significantly weakened, which is due to the nanostructures and residual internal stresses caused by the large deformation of the music steel wire after cold drawing, so that the internal ferrite lamella refinement and dislocation density of the music steel wire increased, and the occurrence of cementite lamella deformation and decomposition and the formation of carbide nanocrystalline particles and part of the amorphous cementite, which is reflected in the music steel wire having a higher tensile strength [22].

**Figure 7:**
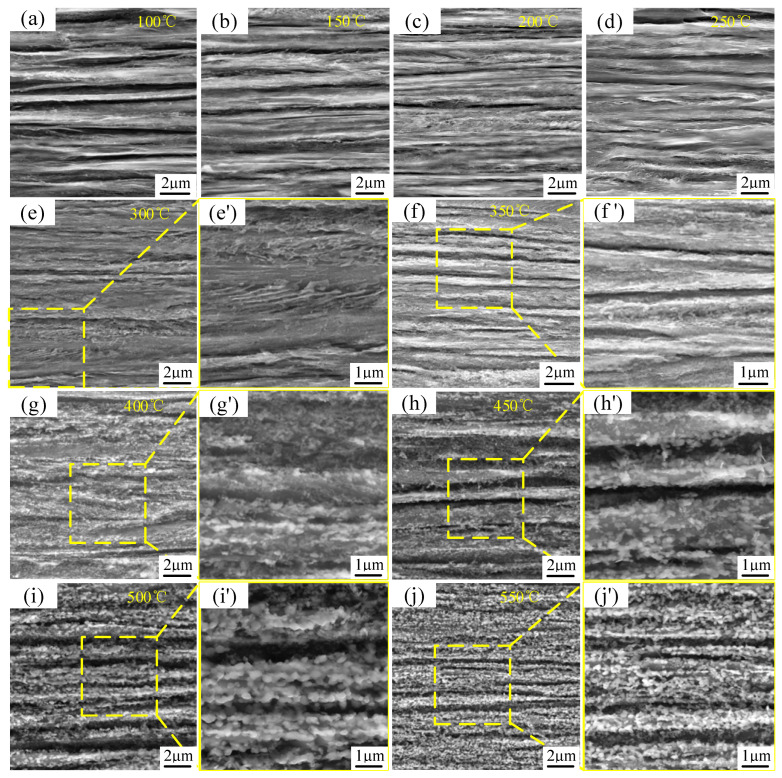
SEM micrographs of the music wires with different annealing treatments: (**a**–**j**) Annealed at 50-degree intervals from 100 °C to 550 °C for 30 min each at a magnification of 20,000 times; (**e′**–**j′**) Localized areas corresponding to 50,000× magnification of each.

Figure 7 presents the SEM images of music steel wire magnified at 20,000× and localized SEM images at 50,000× magnification under varying annealing conditions. The longitudinal microstructure morphology of music steel wire was observed under low-temperature annealing at a conventional temperature for 30 min. It can be observed that, with the increase in annealing temperature, the internal microstructure of music steel wire is gradually spheroidized after annealing at 350 °C, and the internal microstructure of the steel wire is significantly spheroidized when it is annealed at 550 °C.

As shown in Figure 7, the microstructure does not differ much from the original cold-drawn condition after annealing at 150 °C for holding 30 min. Although fragmentation of the cementite flakes can be observed, the lamellar structure is still dominant in the large-strain music steel wire after annealing at 250 °C for holding 30 min (Figure 7d), and the lamellar structure remains stable, there is no spheroidization of the cementite and no recrystallization. A small amount of detached cementite was observed in the annealed wire at 350 °C (Figure 7f). The internal microstructure of the music steel wire is still clearly lamellar-oriented before annealing below 400 °C (Figure 7g) and, after heat treatment at 400 °C for 30 min, substantial changes in the microstructure are observed, accompanied by a notable reduction in lamellar spacing structure of the original lamellar pearlitic phase. In addition, the cementite flakes undergo spheroidization, mainly located in the interlayer spacing junctions. The internal lamellar structure of the music steel wire after annealing from 450 °C (Figure 7h) is still visible in the axial direction, while cell/sub-boundaries were formed in the ferrite, and the cementite flakes started to spheroidize, with the appearance of small-diameter spheroidized carbides. Since the <110> fiber textile structure formed during wire drawing limits further sliding in each lamella to ordinary strain, the compatibility of adjacent grains can only be maintained by bending of the grains toward each other [23], so that the cementite lamellae curl and fragment into numerous short segments or even round grains. This also proves that cementite flakes are capable of plastic deformation and even strain-induced fragmentation [24]. Meanwhile, according to Y.J. Li [25], it was shown that atom redistribution was not observed after annealing at 250 °C for 30 min, and during annealing at 450 °C (Figure 7h), due to the uncertainty of the existence of layered cementite before annealing, there may be interactions between carbon atoms and dislocations, causing carbon atoms dissolved in ferrite to migrate to the pristine carbide. This migration is driven by a reduction in the ferrite/cementite phase boundary driven by a reduction in the ferrite/cementite phase boundary area, resulting in the formation of amorphous cementite crystallization generation and spheroidization of the cementite [2,26]. With increasing temperature, the spheroidization process becomes more pronounced and reaches its maximum at 550 °C (Figure 7j), where the internal microstructure is completely transformed into a distinct spheroidal cementite phase, coexisting with the formation of highly deformed ferrite flakes which recrystallize and agglomerate into larger ferrite grains, with a blurred and almost invisible/cementite phase boundary.

### 3.2. Microstructure Analysis of Music Wire After High-Temperature Annealing

**Figure 8:**
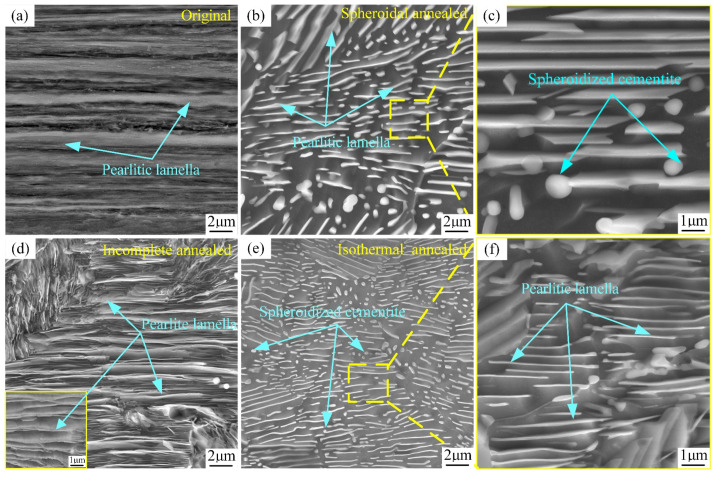
SEM micrographs of the musical steel wires of SWP-B following annealing treatments: (**a**) Original music wire; (**b**) Spheroidal annealed; (**c**) Spheroidal annealed partial enlargement; (**d**) Incomplete annealed; (**e**) Isothermal annealed; (**f**) Isothermal annealed partial enlargement.

Figure 8 depicts the SEM microstructure of SWP-B music steel wire at 20,000× magnification following a high-temperature annealing treatment. The treatment processes of spheroidization annealing (Figure 8b,c), isothermal annealing (Figure 8e,f), and incomplete annealing (Figure 8d) were employed for temperatures exceeding the phase transition point of music steel wire, respectively.

As illustrated in Figure 8a, the microstructure of the music steel wire under 20 k magnification exhibits distinct axial lamellar pearlite spacing, as evidenced by the clear separation of the lamellae. Figure 8b,c depicts the formation of multiple pearlite clusters with varying orientations resulting from the decomposition of eutectic austenite after reaching the spheroidization and annealing temperatures. Figure 8c presents a magnified view of the interior of the clusters. It can be observed that the ferrite lamellae and cementite lamellae are randomly oriented, and the crystal orientations of the ferrite exhibit no strong tendency towards continuity or discontinuity. These are surrounded by minute particles of cementite [27]. Figure 8d illustrates the formation of multiple pearlite clusters with varying orientations, derived from eutectic austenite decomposition following the attainment of an incomplete annealing temperature in the music steel wire. The pearlite lamella spacing within these clusters is considerable, exhibiting no spheroidization. Figure 8e depicts the formation of multiple orientations of pearlite clusters in the music steel wire upon reaching the isothermal annealing temperature through eutectic austenite decomposition. The clusters of internal cementite plates have undergone a transformation, assuming the form of either short rods or spherical particles. The influence of cementite on the deformation of music steel wires is considered to be anisotropic due to the axially aligned laminar structure of the wire [28]. The cementite plate is regarded as a barrier that prevents dislocations from passing through the ferrite, resulting in the deformation direction along the carbide plate being designated as the “soft” orientation of the plastic deformation [29,30]. Conversely, when stress is applied at an angle to the cementite plate, the plate is prone to fracturing, which in turn gives rise to the formation of high-density dislocation entanglement in the ferrite region in close proximity to the fractured cementite. This phenomenon gives rise to the bending of high-density dislocations within the ferrite. As a result, the internal microstructure of the music steel wire, following spheroidization annealing and isothermal annealing of the steel wire, displays a distinctive cementite and ferrite plate-bending phenomenon.

**Figure 9:**
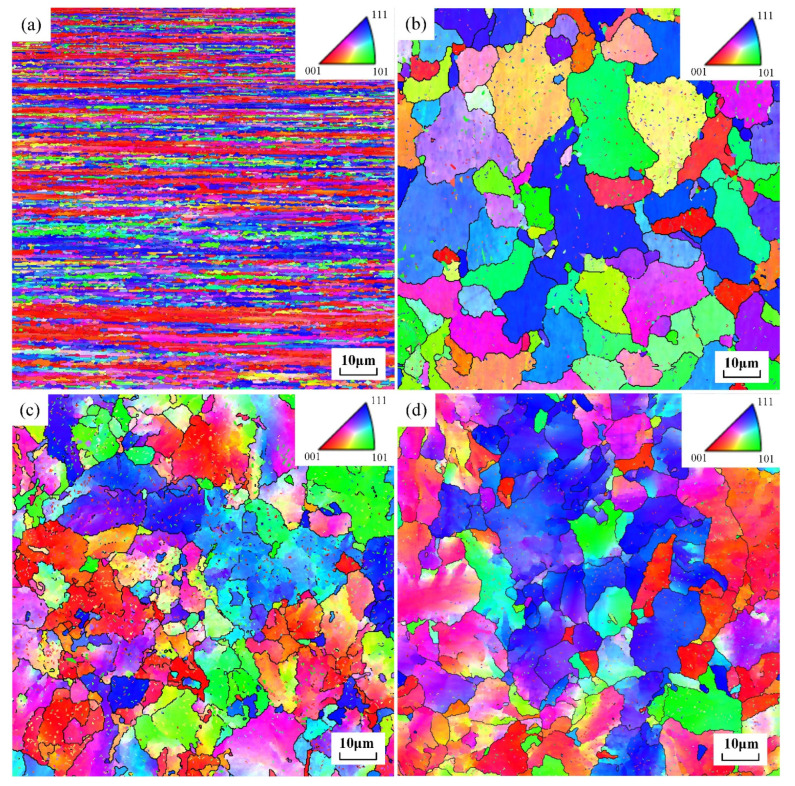
Inverse pole figure (IPF) of ferrite in the pearlite colonies: (**a**) Original music wire; (**b**) Spheroidal annealed; (**c**) Isothermal annealed; (**d**) Incomplete annealed.

**Figure 10:**
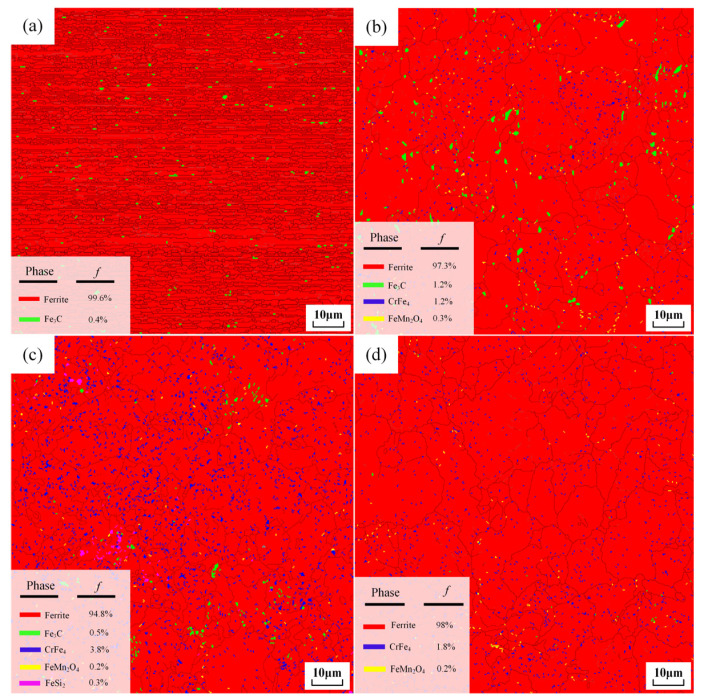
The phase distribution by EBSD: (**a**) Original music wire; (**b**) Spheroidal annealed; (**c**) Isothermal annealed; (**d**) Incomplete annealed.

Figure 9a–d and Figure 10a–d depict the long-axis EBSD color plots and IPF maps and the phase distribution of SWP-B music steel wire subjected to four distinct treatments: original music wire (Figure 9a and Figure 10a), spheroidal annealing (Figure 9b and Figure 10b), isothermal annealing (Figure 9c and Figure 10c), and incomplete annealing (Figure 9d and Figure 10d).

As illustrated in Figure 9a, the original music steel wire exhibits a fiber texture that is nearly parallel to the axial direction, with fine grains displaying an anisotropic structure. Following three distinct high-temperature annealing treatments, the longitudinal cross-section IPF images of the music steel wire, as shown in Figure 9b–d, reveal that, upon annealing of the music steel wire above the phase transition point, there is a notable shift in the internal organization of the material, specifically in the tensile direction of the parallel textile structure segmentation, which generates large-angle grain boundaries and multicrystalline grains. These observations suggest a tendency towards isotropy in each of these structures. Moreover, the recrystallized grains, which diverge from the original organization, exhibit pearlitic lamellar spacing and a drawing direction parallel to the anisotropic state. The latter display a random distribution of pearlitic crystallographic orientation, as the grains continue to grow, resulting in the formation of a fine pearlitic lamellar spacing structure within the grains, as illustrated in Figure 8b–e. The grain size is more uniform following spheroidization annealing. Additionally, a considerable number of minute grain boundaries emerged within the metal wire following isothermal annealing. Furthermore, a combination of minute grain boundaries and larger grains was observed within the steel wire after incomplete annealing. As seen in Figure 10, the content of the cementite phase during spheroidization annealing is the highest under different annealing methods and then decreases continuously, followed by an increase in the content of other precipitated phases.

**Figure 11:**
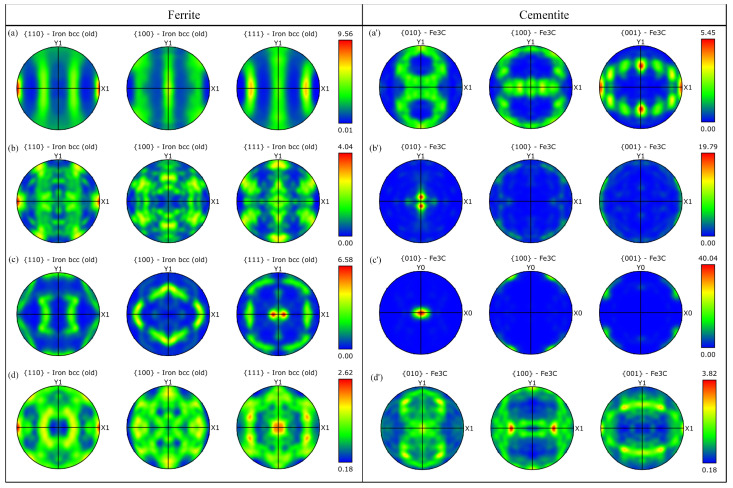
Pole figures of the ferrite and cementite phases of the musical wires with different annealing treatments: Ferrite phase, (**a**) Original music wire; (**b**) Spheroidal annealed; (**c**) Isothermal annealed; (**d**) Incomplete annealed. Cementite phase, (**a′**) Original music wire; (**b′**) Spheroidal annealed; (**c′**) Isothermal annealed; (**d′**) Incomplete annealed.

Figure 11a–d,a′–d′ illustrate the PF plots of the ferrite phase and cementite phases of the original music steel wire, as well as the spheroidal annealing, isothermal annealing, and incomplete annealing treatments. The distribution of the phases can be clearly observed from the PF phase distribution diagrams. Additionally, the evolution of the internal microstructure of the music steel wire deformation texture under different annealing treatment levels is depicted by the polar diagrams of the ferrite and cementite phases.

The strengths of the ferrite <110> fiber texture of the unannealed music steel wire and the spheroidally annealed, isothermally annealed, and incompletely annealed music steel wires are quantified and presented in Figure 11a–d. Figure 11a illustrates that the strength of the ferrite <110> fiber texture of the original music steel wire is 9.56, which corresponds to a markedly robust strength arising from the situation. As the annealing temperature increases, the axial pearlitic lamella spacing inside the steel wire is destroyed, the ferrite phase is decomposed, and the strength of the <110> fiber texture is significantly reduced. This reduction in strength is observed until the incomplete annealing treatment, at which point the strength of the ferrite phase <110> fiber texture decreases to 2.62. Figure 11a′–d′ demonstrate that the formation of spheroidized cementite results in an enhancement of the strength of the <110> phase of cementite. The isothermally annealed steel wire microstructure exhibits the highest strength of the cementite <010> phase, reaching 40.04, which can be attributed to its highest spheroidization rate after isothermal treatment.

**Figure 12:**
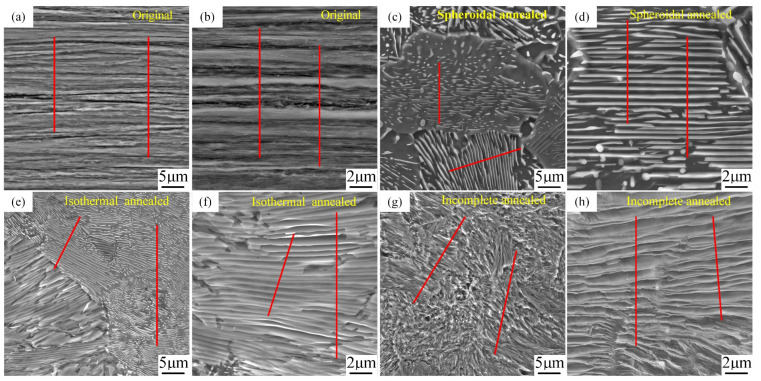
SEM images of pearlite colonies at 10 k times and 20 k times after various annealing treatments: (**a**,**b**) Original music wire; (**c**,**d**) Spheroidal annealed; (**e**,**f**) Isothermal annealed; (**g**,**h**) Incomplete annealed.

Figure 12a–h illustrate the pearlite clusters of unannealed, spheroidally annealed, isothermally annealed, and incompletely annealed steel wires. Given that the music steel wire is a high-carbon steel wire, it follows that the mechanical properties of the high-carbon steel wire and the pearlitic lamellar spacing are inextricably linked. Pearlite is a mixture of ferrite and cementite, and the pearlite organization offers the benefits of high strength and good plasticity [31]. The pearlitic rate serves as a crucial metric for assessing the organization of the steel wire. To ensure accurate measurement of the pearlitic lamellar spacing of the steel wires following different annealing treatments, four groups of pearlitic clusters were selected from each of the four groups visible in the 20k times scanning electron microscope images. The intercept method was employed to ascertain the pearlitic lamella spacing (ILS) [32], with the red line representing an arbitrary intercept line, as illustrated in Figure 12a–h. The measured results are presented in Table 2.

The micrographs obtained via EBSD (Figure 9) demonstrate that the size of the pearlite clusters remains consistent following both spheroidization annealing and isothermal annealing treatments. This is attributed to the comparable heat treatments administered prior to isothermal transformation (Figure 4). Figure 9b–d illustrate that the cluster size diminishes with a reduction in isothermal transformation temperature. The refinement of the pearlite cluster size is typically accompanied by an increase in the number of nucleation sites in the cementite at higher supercooling [33]. The transformation of music steel wires at lower temperatures is distinguished by a reduction in interlayer spacing, which can be attributed to the increase in nucleation sites during supercooling and a concomitant decrease in the diffusion rate. The interlayer spacing of pearlite in SWP-B music steel wire exhibits the smallest value following isothermal annealing treatment. In comparison to the spheroidization annealing heat treatment process at an isothermal temperature of 710 °C, the isothermal annealing process at an isothermal temperature of 600 °C is relatively low (Figure 4b), which results in a reduction in the ability of grain boundaries to move, a slowing down of the growth rate of pearlite, and a refinement of the final pearlite lamella spacing in comparison to the spheroidal annealing [34]. The reduction of pearlitic lamellar spacing results in a slight increase in tensile strength for isothermally annealed music steel wire in comparison to spheroidally annealed wire. This phenomenon can be attributed to the fact when the wire is subjected to an external force, plastic deformation occurs within the ferrite. It is the cementite layer that plays a role in preventing this deformation, and its lamellar spacing represents the maximum slip distance. It can be reasonably deduced that a reduction in lamellar spacing will result in a reduction in slip distance, as well as in ferrite and cementite obstruction. Furthermore, the steel wire will demonstrate an increase in resistance to plastic deformation, which will consequently result in an increase in hardness.

### 3.3. Mechanical Properties Analysis

**Figure 13:**
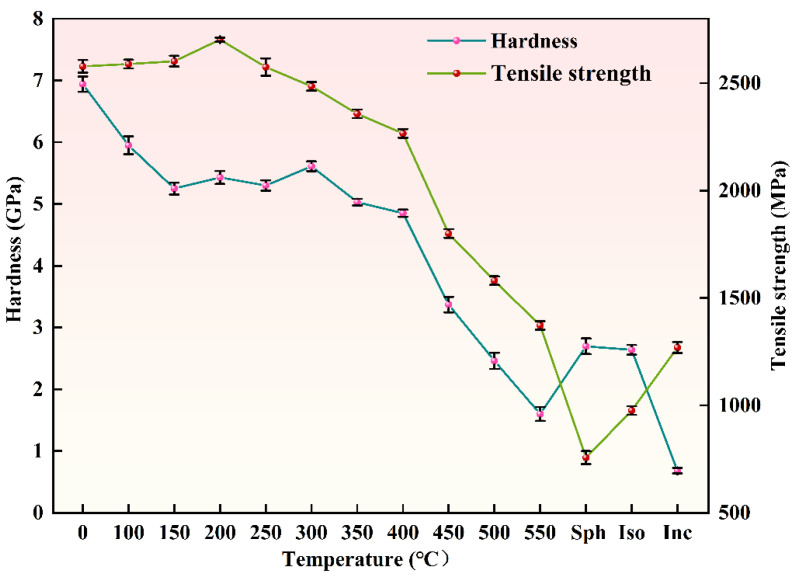
Hardness and tensile strength of music wire after different annealing treatments.

Figure 13 illustrates the variation in nanoindentation hardness and tensile strength of SWP-B musical steel wire following different heat treatments. The microstructure of the SWP-B musical steel wire is predominantly lamellar pearlite. The observed decline in hardness can be attributed to the alteration in the structural composition of pearlite and cementite as a consequence of elevated annealing temperatures.

The cementite present in the music steel wire is capable of withstanding the same degree of plastic strain as the ferrite during cold drawing, despite the inherent brittleness of the cementite itself [27,35,36]. No notable reduction in strength was observed following annealing at 150 °C for 30 min (Figure 13). Instead, a slight increase in strength was observed, reaching a peak of 2.7 GPa after annealing at 200 °C for 30 min. Following annealing and holding at 250 °C for 30 min, a slight decline in strength was observed. However, the level remained at approximately 2.5 GPa, indicating that no significant alterations in the microstructure were apparent. Moreover, it has also been demonstrated that, during annealing at relatively low temperatures (150–300 °C), reprecipitation, nanocrystals, and roughening of severely strained cementite occur [2,4]. Accordingly, the prevailing view in the literature is that the formation of cementite nanocrystals after a period of low-temperature annealing at approximately 250 °C results in wire hardening and a catastrophic deterioration of the torsional toughness of the wire [37,38]. This is evidenced by the fluctuating indentation hardness of the music steel wire between 150 °C and 300 °C as the annealing temperature increases, followed by a continuous decrease, and significant decomposition of the cementite occurs, while the lamellar structure remains dominant [39]. At temperatures exceeding 400 °C, the original layered structure is replaced by a supersaturated ferrite substructure comprising grains [8]. Furthermore, the coarsening of the ferrite substructure grains gradually becomes the dominant structure, exerting control over the strength of the material during subsequent annealing. This is the primary factor responsible for the observed decrease in strength during annealing. While the hardness measurements are predominantly surface-based, the observed decrease in hardness can be attributed to the dissolution of the amorphous mixed cementite structure, which was induced by the large deformation of the drawn music steel wires after annealing at 400 °C for 30 min, resulting in a significant reduction in their surface hardness [40]. It is noteworthy that the spheroidization of cementite begins to predominate during annealing at 400 °C (Figure 7). Following annealing at 450 °C for 30 min, the spheroidization of cementite intensifies, and the hardness of the music steel wires appears to decline, reaching 3.37 GPa (approximately 343 HV), which is about half of the hardness of the unannealed music steel wires, which is 6.94 GPa (approximately 707 HV). The internal lamellar structure of the music steel wire is no longer present after annealing at temperatures up to 550 °C, and the hardness also decreases to 1.6 GPa (approximately 163 HV).

Above the phase transition point, the high percentage of spheroidization of the isothermally spheroidized and isothermally annealed cementite, as well as the presence of the pearlitic lamellar spacing structure (Figure 8b,c), resulted in a slightly higher hardness of the isothermally spheroidized and isothermally annealed microstructure than that of the incompletely annealed microstructure and that of the low-temperature annealed microstructure at 500 °C and 550 °C. In particular, the incompletely annealed microstructure of SWP-B music steel wire exhibits minimal spheroidization (Figure 8d) and, consequently, the material exhibits the lowest recorded hardness value of 0.68 GPa (69 HV).

Figure 13 also demonstrates the correlation between tensile strength and annealing temperature. The tensile strength of the music steel wire at room temperature is 2578 GPa, which indicates that the wire is of a robust and high-strength construction. Following annealing at 200 °C for 30 min, the tensile strength is maintained with a slight increase to 2702 MPa. Following annealing at 250 °C for 30 min, a slight reduction in tensile strength is observed, although it remains at a high level. For annealing temperatures above this threshold, the relationship between tensile strength and annealing temperature is approximately linear, exhibiting a decrease in tensile strength. In high-temperature annealing above the phase transition point, the isothermal annealing microstructure and spheroidal annealing microstructure form large-angle grain boundaries, and their internal cementite spheroidization rate accounts for a significant percentage (Figure 9b,c). Consequently, the tensile strength is significantly reduced. However, in the case of an incompletely annealed microstructure, the internal pearlitic lamellar spacing structure of the grain accounts for a considerable proportion of the grain, and the spheroidization rate is relatively low (Figure 9d), resulting in a tensile strength of 1309 MPa. This is higher than that observed in the microstructure after spheroidal annealing and isothermal annealing.

The observed increase in tensile strength of the steel wire following annealing at temperatures below 200 °C for 30 min can be attributed to either static strain aging during low-temperature annealing or even during the storage of cold-deformed material at room temperature. The increase in wire strain serves to mitigate the effects of low-temperature annealing and subsequent strain aging in large-strain music steel wires. This phenomenon can be attributed to the supersaturation of ferrite with carbon during the cold drawing of large-strain music steel wires and the slightly higher temperatures that occur during the drawing process. These conditions allow for the intense polarization of carbon into individual dislocations in the presence of intense ferrite strain during wire drawing [41]. This effect constrains the number of available sites on the carbon-modified dislocations during static storage at room temperature or annealing at low temperature. Furthermore, given that the formation of the ferrite dislocation cell/substructure is also decorated with carbon, the density of individual dislocations is observed to decrease with continued drawing [25].

Interactions between interstitial solute atoms and dislocations also serve to determine a multitude of mechanical properties inherent to steel [42]. As proposed by Y.J. Li, the formation of the cell/substructure grain in large-strain drawn steel wires after annealing at temperatures above 200 °C is not attributable to recrystallization. Rather, it can be attributed to pronounced recovery of the deformed microstructure. Upon annealing at temperatures above 250 °C, softening commences, accompanied by a pronounced decline in tensile strength. This phenomenon is primarily attributed to the static recovery of the deformed microstructure, manifested in the annihilation, rearrangement, or polygonization of ferrite dislocations [2,43]. Furthermore, additional microstructural modifications during annealing, including spheroidization of cementite, redistribution of alloying elements, recovery of point defects (vacancies and interstitials), and relatively minor coarsening of the cell/substructure grain, may also contribute to a reduction in tensile strength. The application of intense plastic deformation, such as cold drawing, results in the formation of a high dislocation density within the material. The majority of dislocations generated during cold drawing are stored at the interface between ferrite and cementite, where they form cell/substructure grains superimposed on the phase boundary [25]. Concurrently, as a consequence of cementite decomposition, dislocations from adjacent ferrites may converge at the site of the original carburization, thereby forming dipole configurations [44]. In conjunction with the accelerated diffusion process across the phase boundary resulting from severe plastic deformation, the rapid annihilation of dislocation dipoles during annealing becomes a viable phenomenon.

The inhibition of recrystallization in large-deformation SWP-B music wires can be attributed to their fiber texture [45], which reduces the drive for recrystallization and decreases the mobility of grain boundary boundaries, as well as the formation of small-angle grain boundaries. The presence of a pearlitic lamellar spacing microstructure in music steel wires contributes to an increase in strength and hardness compared to wires containing spheroidized cementite. This is attributed to the fact that the latter material exhibits a smaller heterogeneous interfacial area fraction. During annealing at temperatures above 200 °C, the phase boundaries diminished by spheroidized cementite are replenished by the cell/substructure grain, which inhibits dislocation motion. Furthermore, the ferrite cell/substructure grain undergoes continuous coarsening in conjunction with the spheroidization of cementite throughout the annealing process [2]. In the present case, it is challenging to quantify the reduction in tensile strength due to the loss of phase boundary density resulting from the spheroidization of the cementite. It is evident that the softening effect resulting from phase boundary loss is concurrent with the softening effect associated with cell/substructure grain coarsening.

### 3.4. Analysis of Musical Expression of Annealed Musical Wire

**Figure 14:**
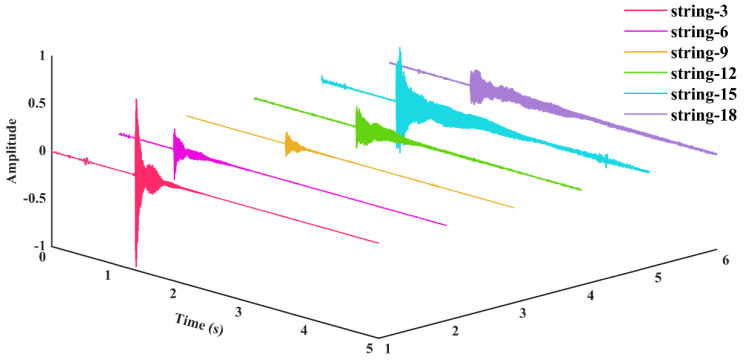
Time domain plot of treble strings 3 and 6, alto string 9, bass strings 12 and 15, and double bass string 18 at standard tones.

**Figure 15:**
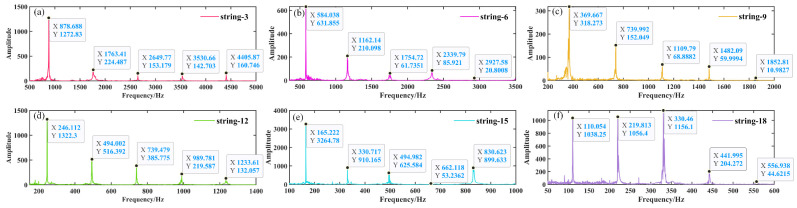
Frequency domain plot of treble strings 3 and 6, alto string 9, bass strings 12 and 15, and double bass string 18 at standard tones. (**a**) Frequency domain plot of string 3; (**b**) Frequency domain plot of string 6; (**c**) Frequency domain plot of string 9; (**d**) Frequency domain plot of string 12; (**e**) Frequency domain plot of string 15; (**f**) Frequency domain plot of string 18.

**Figure 16:**
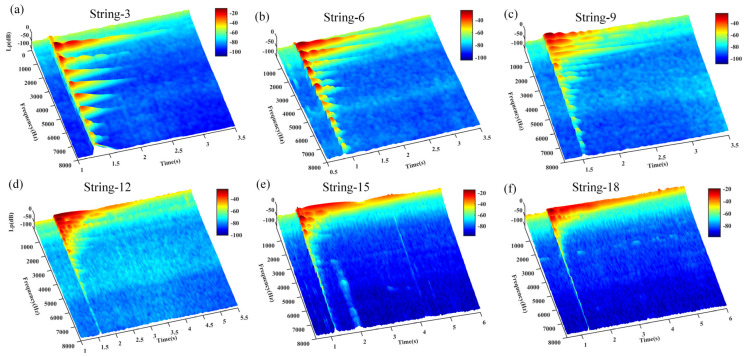
The 3D spectrograms of treble strings 3 and 6, alto string 9, bass strings 12 and 15, and double bass string 18 at standard tones: (**a**) Time and frequency domain plots of string 3; (**b**) Time and frequency domain plots of string 6; (**c**) Time and frequency domain plots of string 9; (**d**) Time and frequency domain plots of string 12; (**e**) Time and frequency domain plots of string 15; (**f**) Time and frequency domain plots of string 18.

**Figure 17:**
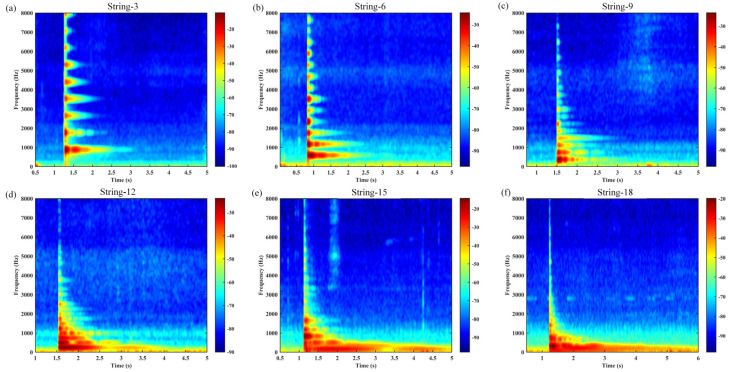
The 2D spectrograms of treble strings 3 and 6, alto string 9, bass strings 12 and 15, and double bass string 18 at standard tones. (**a**) Time and frequency domain plots of string 3; (**b**) Time and frequency domain plots of string 6; (**c**) Time and frequency domain plots of string 9; (**d**) Time and frequency domain plots of string 12; (**e**) Time and frequency domain plots of string 15; (**f**) Time and frequency domain plots of string 18.

Without loss of generality, Figure 14, Figure 15, Figure 16 and Figure 17 illustrate the selected nylon–steel composite wires of the guzheng, which are located on the 3rd string (pitched at 880 Hz) and 6th string (pitched at 493.8 Hz) in the treble region, the 9th string (pitched at 329 Hz) in the middle region, the 12th string (pitched at 220 Hz) and the 15th string (pitched at 146.8 Hz) in the bass region, and the 18th string (pitched at 110 Hz) in the double bass region. The strings are tuned in such a way that they are susceptible to excitation by the sound waves themselves, once they have been tuned. The frequency and time domain plots of the sound waves generated following a Fourier transform are presented herein.

As illustrated in Figure 14, Figure 15, Figure 16 and Figure 17, the guzheng strings are composed of nylon–steel composite wire, which produces discernible vibration signals and a prolonged aftertone. These strings are distinguished by a full and rounded timbre, a purity and softness of tone, brightness, and clarity. As the vibration of the guzheng string is a compound vibration, it exists in two distinct regions, namely the center and one third of the string. This results in the production of a compound tone, which includes both the fundamental frequency and overtones. The low-frequency overtones are of greater amplitude, resulting in a mellower tone that is more suitable for individuals with more sensitive hearing. However, the high-frequency output is less pronounced, and the tone may be perceived as more monotonous. Figure 17 illustrates that the bass strings 12 and 15, in addition to the double bass string 18, exhibit a prolonged sustain and a gradual decay. In contrast, string 9 exhibits a more robust mid-frequency overtone, resulting in a tone that is more rounded and harmonious. In contrast, strings 3 and 6 exhibit augmented high-frequency overtones, which contribute to a brighter and more discernible tone. In the performance, the tone situated in the vicinity of the front yue-shan (a musical instrument part, specifically a horizontal wooden piece used to hold the strings on the forehead of the zither in ethnic musical instruments) is more robust, while the tone in the middle position of the zither code and the front yue-shan is comparatively softer. The entire vibration process of the guzheng’s articulation and the quantity and strength of the overtones generated have become the essential elements that define the guzheng’s distinctive tone. The tone is significantly influenced by the unique characteristics of the guzheng, including a clear and bright soprano, a round and full mid-range, a deep and dense bass, and a long aftertone.

Table 3 illustrates the mechanical property parameters that were evaluated for each of the selected nylon–steel composite wires, in addition to the bare steel wire, the 200 °C annealed steel wire, and the incomplete annealed steel wire.

As illustrated in Table 3, the tensile strength of nylon–steel composite wire with a core steel wire diameter of φ = 0.6 mm, bare steel wire with a diameter of *φ* = 0.6 mm, 200 °C annealed steel wire, and incompletely annealed steel wire is 460 MPa, 2578 MPa, 2702 MPa, and 1269 MPa, respectively. Furthermore, the Young’s modulus is 6.4 GPa, 24.1 GPa, 32.0 GPa, and 7.3 GPa, respectively. Following annealing at 200 °C, the tensile strength of the steel wire reached a maximum of 2.7 GPa. Subsequent low-temperature annealing treatment resulted in an even higher tensile strength of 1.2 GPa in the case of the incompletely annealed steel wire. The enhanced tensile strengths permit a greater degree of inharmonicity in the utilization of the musical steel wire [18,46], thus the steel wire strings that have undergone annealing at 200 °C and the incomplete annealed steel wire strings that have been subjected to high temperatures were selected for the assessment of the musical expression of the strings. The most significant factor influencing the functionality of a string is its stiffness. This term does not refer to the hardness of the string but rather to the external force required to produce a displacement within the string. The material properties and geometry of the string contribute to the overall stiffness of the string [47]. In general, there is a proportional relationship between the stiffness of a material and its Young’s modulus, *E*. Among the properties of a string, stiffness is of particular significance, given the strong correlation between the level of mechanical and physical properties and acoustic parameters. Moreover, the vibrational properties of the string are frequently employed to generate the sound of stringed instruments [7,47,48].

Materials with high stiffness facilitate the transmission of sound waves with greater efficiency, resulting in a more robust and clearer audio output. It is evident that the stiffness of a material may be subject to alteration as a consequence of tensile forces. This alteration is of particular consequence in audio apparatus, as it can influence the velocity of sound wave propagation and the frequency response of the audio [47,49]. To illustrate, 200 °C annealed steel wire (*E* = 24.1 GPa, *TS* = 2702 MPa) and bare steel wire (*E* = 32.0 GPa, *TS* = 2578 MPa) exhibit increased modulus and tensile strength, allowing them to withstand greater stresses under tension. This results in superior tone retention and a richer, more articulate tone due to their capacity to efficiently conduct high-frequency vibrations. The audio spectral clarity and richness of high-frequency harmonics directly influence the musical expression of the instrument [48]. In contrast, softer materials, such as nylon–steel composite wire (*E* = 6.4 GPa, *TS* = 460 MPa) and incomplete annealed steel wire (*E* = 7.3 GPa, *TS* = 1270 MPa), display greater susceptibility to vibration during stretching. This has an impact on the fullness of the sound quality and can result in a reduction in performance at higher pitches, which in turn affects the overall sound expression. Steel wire that has undergone annealing at 200 °C (with a Young’s modulus of 32 GPa) exhibits the highest Young’s modulus, which provides enhanced rigidity and stability. This makes it an optimal choice for generating higher frequencies and harmonics, which contribute to a more precise and clear sound quality. In comparison, incompletely annealed steel wire (with a Young’s modulus of 7.3 GPa) displays diminished rigidity, which gives rise to instability in its high-frequency expression and a mellifluous, warm tone that may be more conducive to specific applications. However, this is frequently accompanied by a reduction in volume and loudness.

**Figure 18:**
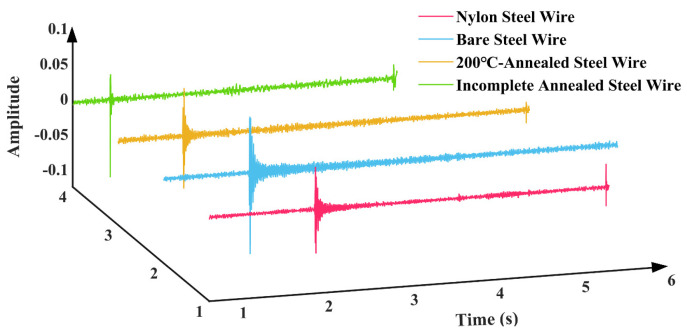
Time domain plot of various string materials after Fourier transform of the A6 tone (standard fundamental frequency of 1760 Hz) played under plucking.

**Figure 19:**
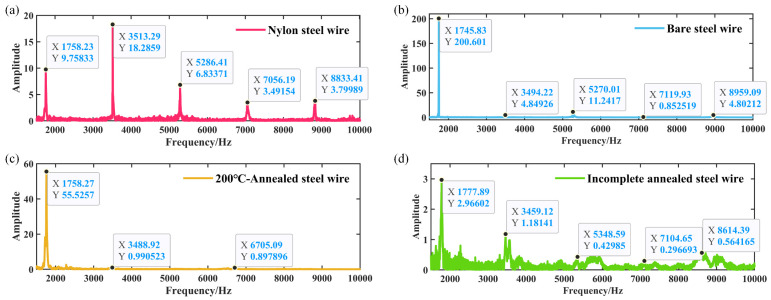
Frequency domain representation of various string materials after Fourier transform of the A6 tone (standard fundamental frequency of 1760 Hz) played under plucking. (**a**) Frequency domain plot of nylon–steel composite wire; (**b**) Frequency domain plot of bare steel wire; (**c**) Frequency domain plot of 200 °C annealed bare steel wire; (**d**) Frequency domain plot of incompletely annealed bare steel wire.

**Figure 20:**
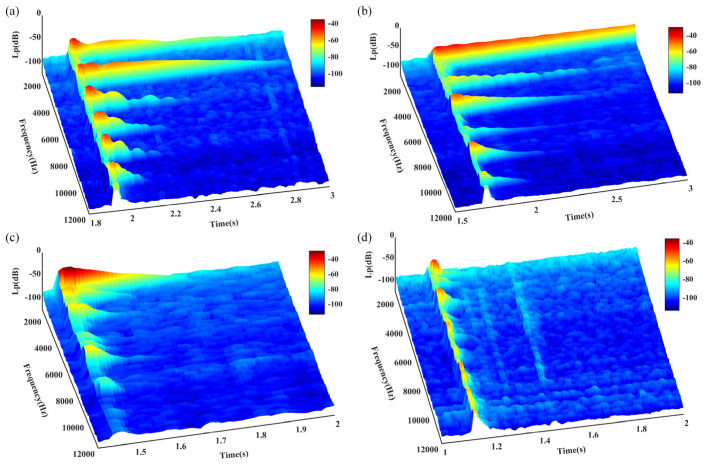
The 3D spectrograms of each string when played under excitation of an A6 tone (standard fundamental frequency of 1760 Hz): (**a**) Time and frequency domain plots of nylon–steel composite wire; (**b**) Time and frequency domain plots of bare steel wire; (**c**) Time and frequency domain plots of 200 °C annealed bare steel wire; (**d**) Time and frequency domain plots of incompletely annealed bare steel wire.

Figure 18, Figure 19 and Figure 20 illustrate the time domain, frequency domain plots, as well as the 3D spectrograms of four different string materials played under excitation for the A6 tone (standard fundamental frequency of 1760 Hz) after Fourier transformations for comparison.

Table 4 presents the frequencies (amplitudes) of the first five harmonics of the A6 tone when played by the four wires that underwent distinct treatments.

As illustrated in Figure 19 and Figure 20 and Table 4, musical wires composed of different materials demonstrate disparate frequency shifts between the various harmonic orders [50]. The first harmonic (fundamental frequency) exhibits a range of approximately 1745 Hz to 1777 Hz, with the highest frequency (1777 Hz) observed in the incompletely annealed steel wire, resulting in a brighter tone. The second harmonic exhibits a relatively narrow frequency range, with the nylon–steel composite wire (3513 Hz) demonstrating superior stability compared to the other materials, indicative of enhanced frequency retention. The spectra of the various wire material distributions demonstrate that the annealing treatment has a considerable impact on the harmonic frequencies [13]. In particular, the third harmonic lacks data for the bare steel wire annealed at 200 °C. However, the nylon–steel composite wire (5286 Hz) and incomplete annealed steel wire (5348 Hz) exhibit higher frequencies, indicating that both may be more effective in high-frequency timbres. Additionally, the fourth and fifth harmonics demonstrate a notable variation in frequency. The steel wire annealed at 200 °C (6705 Hz) exhibited relatively low frequencies at the fourth harmonic, which may contribute to a more profound aftertone. The amplitudes of the harmonics exhibited variability across the different materials. The amplitude characteristics of the bare steel wire and the nylon–steel composite wire are more pronounced in the higher harmonics, particularly the 5th harmonic. This may result in a richer tone and improved layering. The amplitude of the incomplete annealed wire is lower (8614 Hz), which affects the roundness and subtlety of the tone.

Figure 19 demonstrates that the steel wire that has undergone incomplete annealing displays a greater number of harmonics and a less discernible frequency amplitude. Upon the application of an excitation, the stabilization of the fundamental frequency of the nylon–steel composite wire occurs at a slower rate, while the stabilization of the fundamental frequency of the bare steel wire occurs at a faster rate. In conjunction with the Young’s modulus test, the nylon–steel composite wire and the incompletely annealed steel wire exhibited the lowest Young’s modulus value among all the strings, indicating that these materials possess relatively low stiffness and high deformability. This resulted in an instability and a less uniform vibration pattern when the strings were subjected to playing stimulation. Furthermore, the fundamental frequency demonstrated a prolonged stabilization period. The nylon–steel composite wire displays a greater richness of frequency components, a relatively weaker presence of high frequencies, and a notable prominence of spectral peaks in the low-frequency range. The fundamental frequency (1758 Hz) is robust and exhibits a gradual decay, while its harmonics (3513 Hz, 5286 Hz, 7056 Hz, 8833 Hz) are somewhat diminished and decay more rapidly, allowing for clear differentiation between scales. The timbre of nylon–steel composite wires is typically mellifluous with a warm quality. The phase response of these strings may result in relatively weak volume peaks at specific frequencies. These frequencies are distinguished by lower energy in the high frequencies and a more even distribution of harmonics, often with a greater proportion of low-frequency components. This can be attributed to the enhanced richness and layering of the string’s timbre resulting from the wrapping of the nylon and copper wires, which also increases the durability and abrasion resistance of the strings while maintaining pitch stability and reducing intonation fluctuations [51].

In comparison to nylon–steel composite wire, bare steel wire displays enhanced stiffness, which results in a diminished amplitude and concentrated energy in the lower-frequency region. The fundamental frequency component in the spectrum is markedly amplified, accompanied by a more pronounced and powerful high-frequency component. Furthermore, the harmonics are more pronounced and robust, contributing to a brighter and clearer tone with a wider frequency distribution. The resonance effect and transient response of bare steel wire are typically more pronounced, with the potential for additional high-frequency peaks to appear within the frequency spectrum. Bare steel wires typically exhibit higher power and a more pronounced volume than nylon–steel composite wires and 200 °C annealed steel wires under the same playing force. This characteristic results in the generation of more penetrating acoustic effects. The spectral characteristics of 200 °C annealed steel wire are analogous to those of bare steel wire, albeit with a diminution in harmonics and a slight attenuation of the high-frequency range. The fundamental frequency and its harmonic energy are diminished, resulting in a less robust sound than that of ordinary steel wire strings. The initial stages of harmonic attenuation are accelerated, and the overall timbre is reminiscent of that nylon–steel composite wires. Nevertheless, the distinctive attributes of bare steel wire characteristics remain discernible. The spectrum of the incompletely annealed steel wire exhibits the most moderate characteristics. The application of high-temperature annealing can facilitate the release of internal stress, thereby enhancing the resonance and high-frequency gain. The spectrum displays a substantial high-frequency component and a complex harmonic structure. However, the energy is low and the sound quality is relatively poor, with a noticeable decrease in high-frequency power.

**Figure 21:**
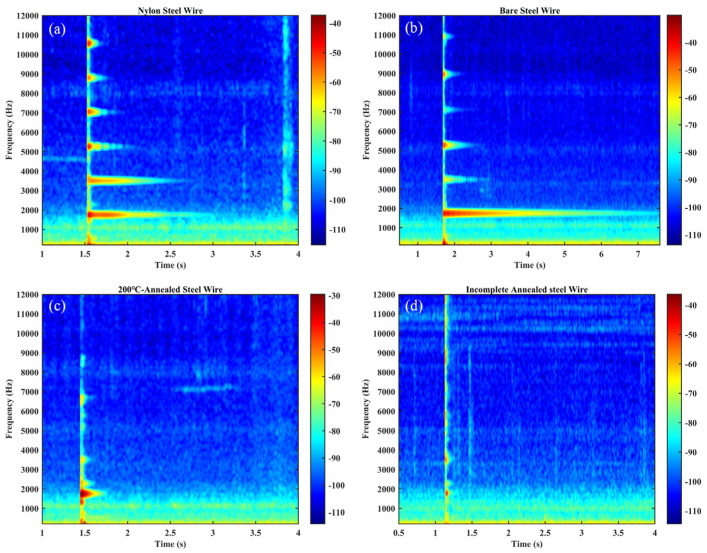
The 2D spectrograms of the acoustic signals produced by each string under plucking excitation: (**a**) Time–frequency plot of nylon wire composite wire; (**b**) Time–frequency plot of bare steel wire; (**c**) Time–frequency plot of 200 °C annealed steel wire; (**d**) Time–frequency plot of incompletely annealed steel wire.

Figure 21 illustrates the time–frequency plots of the acoustic signals generated by each string of the aforementioned guzheng under plucking excitation following distinct heat treatments. These plots demonstrate the regularity of the changes in harmonic components over time.

Table 5 presents the calculated decay times of the fundamental frequency harmonic components of the acoustic signals generated during the vibration of four typical guzheng strings under excitation.

As illustrated in Figure 21 and Table 5, the duration of the intervals for all four strings of the guzheng exhibits variability to varying degrees. Consequently, the duration of the resulting sound signals is also subject to alteration. The fundamental wave components of all four strings of the guzheng reach their peak values with remarkable swiftness after the application of the plucking force and then undergo a rapid decay until they cease to exist [12].

The various categories of wires exhibited notable differences in their respective decay times. The bare steel wire exhibited the longest decay time (319.7 ms), while the incompletely annealed steel wire demonstrated a markedly shorter decay time of 2.3 ms, indicating a rapid decay of vibration energy. These findings suggest that there are notable differences in vibration retention among various materials. This phenomenon is associated with the impact of attenuation on sound vibration, which is contingent upon the specific microstructure of the material in question [52]. The decay time of the steel wire that has undergone a 200 °C annealing process is 41.6 ms, which indicates that it is better able to retain its acoustic properties. In contrast, the nylon–steel composite wire shows better sound retention in practical applications (96.5 ms), indicating that the material properties of the incompletely annealed steel wire are more favorable than those of the aforementioned wire, but not as optimal as those of the bare steel wire. The incorporation of a winding layer into the latter results in an increase in the weight of the string, the tension of the string, and the vibration characteristics of the steel wire core. This ensures a suitable amplitude and markedly improves the sound quality of the string.

The rest of the nylon–steel composite wire is shorter than that of bare steel wire. The material has a more readily dissipated sound, a relatively low sound beta value, a smaller volume, and is suitable for the expression of delicate, soft, and lyrical music. However, the material’s softness constrains the emotional richness that it is capable of expressing. The fundamental frequency of the initial excitation exhibits enhanced stability, however, it is susceptible to fluctuations resulting from fingering. The fundamental frequency stabilization of bare steel wire occurs more rapidly, allowing it to quickly reach a stable state. The pitch accuracy is higher, with a decibel value that is greater, a volume that is more pronounced, rich harmonics, an aftertaste that is longer, a relatively long decay time, and a slower auditory disappearance. Bare steel wire is capable of expressing a wide range of emotions, rendering it an optimal selection for fast-paced, high-intensity musical compositions. The bright sound quality of this instrument allows for the clear conveyance of a multitude of musical emotions.

Table 5 lends further support to the hypothesis that the frequency of annealed steel wire at 200 °C is analogous to that of nylon–steel composite wire, with the latter displaying a more pronounced first-order frequency (1758 Hz). However, the first harmonic decays more rapidly, resulting in a notable decline in the amplitude of high-order frequencies. Although these changes may be discernible to the human ear, the resulting lack of rhythm and lack of tone, along with the loss of musical beauty, are less perceptible. The microstructure of the 200 °C annealed material is more uniform, which results in enhanced energy propagation characteristics, improved mechanical properties, and a fuller, relatively warm tone. This renders it more conducive to the expression of emotionally rich music. The annealing process has the effect of reducing internal stresses, increasing the toughness and elasticity of the string, and enhancing its responsiveness when played. The annealing process alters the crystalline structure of the material, resulting in a refinement of the grain structure. This improvement in the material’s structure enhances the musical expression and sound quality of the string. A more uniform vibration results from a superior microstructure, which in turn gives rise to a richer harmonic characteristic and an enhanced overall acoustic effect. Consequently, the frequency of incompletely annealed steel wire is relatively high, and the amplitude is significantly lower in the low- and middle-frequency bands. This may result in a lack of warmth in the fundamental tone, rendering it suitable for the expression of certain high-frequency characteristics. Nevertheless, there are some limitations in regard to emotional expression.

## 4. Conclusions

In this study, SWP-B music wire was subjected to a series of heat treatments at varying temperatures, including low-temperature annealing, spheroidal annealing, isothermal annealing, and incomplete annealing. Subsequently, the evolution of the wire’s microstructural properties was examined through a combination of microstructure analysis techniques. This study aimed to investigate the effect of microstructural evolution on the musical expression of SWP-B music wire. The following conclusions can be drawn:

(1) A low-temperature annealing process at 200 °C for 30 min can facilitate the formation of carbon atoms at the sub-boundaries of significant bias polymerization in music steel wire. This is accomplished by reducing the interfacial energy and stabilizing the sub-boundaries, which can then function as a barrier for dislocation movement. Moreover, the precipitation of fine interstitial carbides can impede dislocation movement, thereby conferring an additional strengthening effect on the wire. The tensile strength of SWP-B piano wire was increased from 2578 MPa to 2702 MPa.
(2) As the temperature of the low-temperature annealing process is increased, the precipitation of some intermediate carbides (ε-Fe_3_C) occurs at the interfacial position of adjacent ferrite lamellae and in the ferrite matrix. Following low-temperature tempering at 450 °C, notable precipitation changes were observed for the elements Cr, Mn, and Si.
(3) Subsequent to spheroidizing, isothermal, and incomplete annealing treatments, the structure of SWP-B music wire undergoes a transformation from anisotropic to isotropic, exhibiting a near-random texture. This process results in a notable decline in both hardness and tensile strength. The strength of the ferrite phase <110>//ND and the cementite phase <010>//ND exhibited a gradual decline with the increase in annealing temperature.
(4) Nylon–steel composite wire strings are suitable for soft-toned musical instruments, although further improvement may be necessary in order to achieve optimal high-frequency characteristics. The annealed steel wires, subjected to a 30 min treatment at 200 °C, display superior frequency retention and sound expression characteristics in comparison to other materials. Consequently, they are well-suited for musical instruments that require high-quality tone and high-frequency characteristics. It has been demonstrated that incomplete annealing and other high-temperature annealing treatments result in a notable reduction in both tensile strength and Young’s modulus of steel wire. Such alterations frequently result in unstable frequency changes, which have a detrimental impact on the tone. Furthermore, the resulting tone and performance are inadequate for the high standards required for musical instrument manufacturing.

## Figures and Tables

**Table 1:** Chemical Composition of High Carbon Music Wire of SWP-B (wt.%).

Element	Fe	C	Si	Cr	Mn	Cu	P	S	Al
Music wire	Bal	0.781	0.21	0.06	0.67	0.04	0.001	0.004	0.005

**Table 2:** The pearlitic lamella spacing measurements of music wire after various annealing treatments.

	Lamella Spacing (μm)				
	1	2	3	4	Average Value
Original music wire	0.75966	0.86182	0.85458	0.87536	0.83786
Spheroidal annealed	0.37564	0.38747	0.37470	0.36415	0.37549
Isothermal annealed	0.22160	0.26875	0.26760	0.26469	0.25566
Incomplete annealed	0.53573	0.53024	0.56706	0.57031	0.55084

**Table 3:** Mechanical performance parameters of music wires after varying annealing treatment conditions.

Music Wires	Diameter/mm	Modulus of Elasticity/(GPa)	Tensile Strength/(MPa)
Nylon–steel composite wire	1.75	6.4 ± 0.7	460 ± 24
Bare steel wire	0.6	24.1 ± 1.3	2578 ± 37
200 °C annealed bare steel wire	0.6	32.0 ± 1.8	2702 ± 53
Incompletely annealed bare steel wire	0.6	7.3 ± 1.4	1270 ± 48

**Table 4:** Amplitudes of the first five harmonics of the A6 tone played on four different strings.

	Nylon–Steel Composite Wire	Bare Music Wire	200 °C Annealed Bare Music Wire	Incompletely Annealed Bare Music Wire
1st harmonic	1758 Hz (9.75)	1745 Hz (200.6)	1758 Hz (55.52)	1777 Hz (2.96)
2nd harmonic	3513 Hz (18.28)	3494 Hz (4.85)	3488 Hz (0.99)	3459 Hz (1.18)
3rd harmonic	5286 Hz (6.83)	5270 Hz (11.24)	-	5348 Hz (0.43)
4th harmonic	7056 Hz (3.49)	7119 Hz (0.85)	6705 Hz (0.89)	7104 Hz (0.30)
5th harmonic	8833 Hz (3.80)	8961 Hz (4.93)	-	8614 Hz (0.56)

Note: The audio signal is processed by Fourier transform to ensure the amplitude value is relative and dimensionless.

**Table 5:** Time required for the 1st harmonic of each string to decay to 10% of its peak value.

	Nylon Steel Wire	Bare Steel Wire	200 °C Annealed Steel Wire	Incompletely Annealed Steel Wire
Time	96.5 ms	319.7 ms	41.6 ms	2.31 ms

## Data Availability

The original contributions presented in this study are included in the article. Further inquiries can be directed to the corresponding authors.
